# Validation of a screening test for alcohol use, the Russian Federation

**DOI:** 10.2471/BLT.20.273227

**Published:** 2021-03-19

**Authors:** Maria Neufeld, Jürgen Rehm, Anna Bunova, Artyom Gil, Boris Gornyi, Pol Rovira,, Jakob Manthey, Elena Yurasova, Svetlana Dolgova, Bulat Idrisov, Marina Moskvicheva, Galina Nabiullina, Olga Shegaym, Irina Zhidkova, Zukhra Ziganshina, Carina Ferreira-Borges, & the 2019/2020 RUS-AUDIT Collaborators & the RUS-AUDIT Project Advisory Board

**Affiliations:** a World Health Organization European Office for Prevention and Control of Noncommunicable Diseases, Leontyevsky Pereulok 9, 125009 Moscow, Russian Federation.; b Institute for Mental Health Policy Research, Centre for Addiction and Mental Health, Toronto, Canada.; c National Medical Research Center for Therapy and Preventive Medicine of the Ministry of Health of the Russian Federation, Moscow, Russian Federation.; d Institute for Leadership and Health Management, I.M. Sechenov First Moscow State Medical University, Moscow, Russian Federation.; e Program on Substance Abuse, Public Health Agency of Catalonia, Barcelona, Spain.; f Institute for Clinical Psychology and Psychotherapy, Technische Universität, Dresden, Germany.; g World Health Organization Office in the Russian Federation, Moscow, Russian Federation.; h Vologda City Policlinic, Vologda, Russian Federation.; i Moscow Institute of Physics and Technology, Moscow, Russian Federation.; j Department of Public Health and Healthcare, South Ural State Medical University, Chelyabinsk, Russian Federation.; k Center for Medical Prevention, Astrakhan, Russian Federation.; l Center for Medical Prevention, Tomsk, Russian Federation.; m Amur Regional Center for Preventive Medicine, Blagoveshchensk, Russian Federation.; n Institute of Management, Economics and Finance, Kazan Federal University, Kazan, Russian Federation.

## Abstract

**Objective:**

To validate a Russian-language version of the World Health Organization’s Alcohol Use Disorders Identification Test (AUDIT).

**Methods:**

We invited 2173 patients from 21 rural and urban primary health-care centres in nine Russian regions to participate in the study (143 declined and eight were excluded). In a standardized interview, patients who had consumed alcohol in the past 12 months provided information on their sociodemographic characteristics and completed the Russian AUDIT, the Kessler Psychological Distress Scale and the Composite International Diagnostic Interview to identify problem drinking and alcohol use disorders. We assessed the feasibility of administering the test, its internal consistency and its ability to predict hazardous drinking and alcohol use disorders in primary health care in the Russian Federation.

**Findings:**

Of the 2022 patients included in the study, 1497 were current drinkers with Russian AUDIT scores. The test was internally consistent with good psychometric properties (Cronbach’s *α* : 0.842) and accurately predicted alcohol use disorders and other outcomes (area under the curve > 75%). A three-item short form of the test correlated well with the full instrument and had similar predictive power (area under the curve > 80%). We determined sex-specific thresholds for all outcomes, as non-specific thresholds resulted in few women being identified.

**Conclusion:**

With the validated Russian AUDIT, there is no longer a barrier to introducing screening and brief interventions into primary health care in the Russian Federation to supplement successful alcohol control policies.

## Résumé

**Objectif:**

Valider une version russe du test AUDIT (Alcohol Use Disorders Identification Test) de l'Organisation mondiale de la Santé.

**Méthodes:**

Nous avons invité 2173 patients originaires de 21 centres de soins de santé primaires répartis dans neuf régions du pays, tant en milieu rural qu'urbain, à participer à cette étude (143 ont refusé et huit ont été exclus). Lors de chaque entretien standardisé, les patients ayant consommé de l'alcool au cours des 12 derniers mois ont fourni des informations sur leurs caractéristiques sociodémographiques et complété l'AUDIT russe, l'Échelle de détresse psychologique de Kessler ainsi que le Questionnaire composite international pour le diagnostic afin d'identifier les problèmes de boisson et les troubles liés à l'abus d'alcool. Nous avons évalué la faisabilité du test, sa cohérence interne et sa capacité à prédire toute consommation dangereuse ou trouble lié à l'abus d'alcool dans le cadre des soins de santé primaires en Fédération de Russie.

**Résultats:**

Sur les 2022 patients inclus dans l'étude, 1497 se sont vus octroyer un score par l'AUDIT russe. Le test a fait preuve de cohérence interne et affiché de bonnes propriétés psychométriques (Cronbach *α*: 0,842). Il a également prédit avec justesse les troubles liés à l'abus d'alcool, mais aussi d'autres conséquences (aire sous la courbe > 75%). Une version plus courte du test, comportant seulement trois éléments, s'est bien accordée avec l'instrument complet et offrait une valeur prédictive similaire (aire sous la courbe > 80%). Pour l'ensemble des observations, nous avons déterminé des seuils en fonction du genre car dans le cas contraire, le nombre de femmes identifiées était peu élevé.

**Conclusion:**

Grâce à la validation du test AUDIT russe, aucun obstacle ne subsiste en matière de dépistage et d'intervention rapide dans les centres de soins de santé primaires de la Fédération de Russie. Ces derniers peuvent ainsi s'ajouter aux politiques efficaces de lutte contre l'alcoolisme.

## Resumen

**Objetivo:**

Validar una versión en ruso del Test de Identificación de Trastornos debidos al Consumo de Alcohol (AUDIT, por sus siglas en inglés) de la Organización Mundial de la Salud.

**Métodos:**

Invitamos a participar en el estudio a 2.173 pacientes de 21 centros de atención primaria rurales y urbanos de nueve regiones rusas (143 se negaron y ocho fueron excluidos). En una entrevista estandarizada, los pacientes que habían consumido alcohol en los últimos 12 meses proporcionaron información sobre sus características sociodemográficas y completaron el AUDIT ruso, la Escala de malestar psicológico de Kessler y la Entrevista Diagnóstica Internacional Compuesta para identificar el consumo problemático de alcohol y los trastornos por consumo de alcohol. Se evaluó la viabilidad de la administración de la prueba, su consistencia interna y su capacidad para predecir el consumo peligroso de alcohol y los trastornos por consumo de alcohol en los centros de atención primaria de la Federación Rusa.

**Resultados:**

De los 2.022 pacientes incluidos en el estudio, 1.497 eran consumidores de alcohol con puntuaciones en el AUDIT. La prueba demostró consistencia interna con buenas propiedades psicométricas (Cronbach α : 0,842). También predijo con precisión los trastornos por abuso de alcohol, pero también otras consecuencias (área bajo la curva > 75%). Una versión más corta de la prueba con solo tres ítems se correlacionó bien con el instrumento completo y ofreció un valor predictivo similar (área bajo la curva > 80%). Se determinaron umbrales específicos por sexo para todos los resultados, ya que los umbrales no específicos identificaron a pocas mujeres.

**Conclusión:**

Con la validación del AUDIT ruso, ya no existe ningún obstáculo para el cribado y la intervención temprana en los centros de atención primaria de la Federación Rusa. Estas pueden añadirse a las políticas eficaces de control del consumo de alcohol.

## ملخص

الغرض التصديق على كشف اضطرابات تعاطي الكحول (AUDIT) التابع لمنظمة الصحة العالمية والصادر باللغة الروسية.

الطريقة قمنا بدعوة 2173 مريضًا من 21 مركزًا ريفيًا وحضريًا للرعاية الصحية الأولية في ثماني مناطق روسية للمشاركة في الدراسة (قام 143 مريضًا بالرفض، وتم استبعاد ثمانية). في مقابلة شخصية قياسية، قام المرضى الذين تناولوا الكحول خلال الاثني عشر شهرًا الماضية، بتقديم معلومات عن خلفياتهم الاجتماعية والسكانية، وأكملوا اختبار AUDIT الروسي، ومقياس كيسلر للضغط النفسي، والمقابلة الشخصية التشخيصية الدولية المركبة لتحديد مشاكل الشرب واضطرابات تعاطي الكحول. قمنا بتقييم جدوى إجراء الاختبار، واتساقه الداخل وقدرته على توقع الاضطرابات الخطرة للشرب وتعاطي الكحول في الرعاية الصحية الأولية في الاتحاد الروسي.

النتائج من بين 2022 مريضًا مشمولًا في الدراسة، حصل 1497 من متعاطيي الكحوليات في اختبار AUDIT الروسي على درجة. كان الاختبار متسقًا داخليًا مع الخصائص النفسية الجيدة (Cronbach *α * :0.842)، وتم بدقة توقع اضطرابات تعاطي الكحول والنتائج الأخرى (المنطقة الواقعة تحت المنحنى> 75%). يرتبط نموذج الاختبار ثلاثي العناصر بشكل جيد مع الأداة الكاملة، كما أن له قوة تنبؤية مماثلة (المنطقة الواقعة تحت المنحنى > 80%). قمنا بوضع محددات خاصة بالجنس لجميع النتائج، حيث أدت المحددات غير المحددة إلى اكتشاف عدد قليل من النساء.

الاستنتاج مع استخدام اختبار AUDIT الروسي المصدق عليه، لم يعد هناك عائق أمام طرح اختبار الفحص والتدخلات السريعة في الرعاية الصحية الأولية في الاتحاد الروسي لتكملة السياسات الناجحة لمكافحة الكحول.

## 摘要

**目的:**

旨在确认俄语版世界卫生组织酒精使用障碍识别测试 (AUDIT)。

**方法:**

我们从俄罗斯 9 个地区的 21 个城乡初级卫生保健中心邀请了 2173 名患者(其中 143 例谢绝参加,8 例被排除在外)参与本次调查。在一次标准化访谈中,在过去 12 个月内曾饮酒的患者提供了其社会人口统计学特征相关信息,并完成了俄罗斯 AUDIT、凯斯勒心理困扰量表和复合性国际诊断访谈表,以确定是否存在酗酒和酒精使用障碍。我们评估了俄罗斯联邦初级卫生保健系统实施该项测试的可行性、其内部一致性以及预测危险饮酒和酒精使用障碍的能力。

**结果:**

在参与本次调查的 2022 名患者中,1497 名饮酒者的俄罗斯 AUDIT 得分为。该测试与良好的心理测量特性具有内部一致性(克朗巴哈系数*a*:0.842),并且可以准确预测酒精使用障碍和其他结果(曲线下面积 > 75%)。该测试的三项简单检验项目与整个量表呈正相关,并且具有相似的预测能力(曲线下面积 > 80%)。我们针对所有结果确定了指定性别的阈值,因为若未确定指定阈值,则会导致仅可识别极少数的女性。

**结论:**

通过使用经确认的俄罗斯 AUDIT,俄罗斯联邦初级卫生保健系统在引入筛查测试和简要干预措施方面不再存在障碍,从而可有效促进酒精控制政策的成功实施。

## Резюме

**Цель:**

Провести валидацию русскоязычной версии теста Всемирной организации здравоохранения для выявления расстройств, обусловленных употреблением алкоголя (AUDIT).

**Методы:**

Для участия в исследовании были приглашены 2173 пациента из 21 сельского и городского центра первичной медико-санитарной помощи в девяти регионах России (143 пациента отказались, а 8 были исключены из исследования).В стандартизированном интервью пациенты, употреблявшие алкоголь в течение последних 12 месяцев, предоставляли информацию о своих социально-демографических характеристиках и проходили российский тест AUDIT, тесты по шкале психологического дистресса Кесслера и составное международное диагностическое интервью для выявления проблемного употребления алкоголя и расстройств, связанных с употреблением алкоголя. Авторы оценили целесообразность проведения теста, его внутреннюю согласованность и способность предсказывать чрезмерное употребление алкоголя и расстройства, связанные с употреблением алкоголя, в рамках первичной медико-санитарной помощи в Российской Федерации.

**Результаты:**

Из 2022 пациентов, включенных в исследование, 1497 употребляли алкоголь и набрали баллы в российском тесте AUDIT. Тест был внутренне согласован, обладал хорошими психометрическими характеристиками (Кронбах *α*: 0,842) и точно предсказывал расстройства, связанные с употреблением алкоголя, и другие последствия (площадь под кривой > 75%). Краткая форма теста из трех вопросов соответствовала полной форме и обладала аналогичной прогностической значимостью (площадь под кривой > 80%). Мы определили пороговые значения, зависящие от пола, для всех последствий, поскольку пороговые значения, не зависящие от пола, приводили к выявлению слишком малого количества женщин.

**Вывод:**

Валидированный российский тест AUDIT устраняет препятствия для внедрения скрининга и краткосрочных вмешательств в услуги первичной медико-санитарной помощи в Российской Федерации в дополнение к успешной политике по борьбе с употреблением алкоголя.

## Introduction

In September 2018, the World Health Organization (WHO) released SAFER, a new initiative and technical package outlining five high-impact strategies that can help governments to reduce the harmful use of alcohol and related health, social and economic consequences.^1^ One of these strategies is to “facilitate access to screening, brief interventions and treatment”, which is enabled by the Alcohol Use Disorders Identification Test (AUDIT), a simple 10-item test.^2^^,^^3^ The test is the most successful screening instrument for assessing an individual’s hazardous and harmful use of alcohol and for alcohol use disorders worldwide. The term harmful use is used as a diagnostic code (F10.1) in the *International classification of diseases and related health problems, 10th revision* (ICD-10) in the section: mental and behavioural disorders due to psychoactive substance use.^4^ The term hazardous use is a non-diagnostic term which denotes a pattern of alcohol consumption carrying a risk of harmful consequences to the drinker.^2^ Developed by WHO, the AUDIT was primarily intended for screening purposes in primary health care to identify individuals with the above-mentioned drinking patterns and potential alcohol dependence. Today, the AUDIT, or its short form, the AUDIT-C (three-item form),^5^ is being used as the main screening instrument in these settings globally. Screening and brief interventions have become a standard part of any comprehensive alcohol policy, especially since WHO launched the SAFER initiative.^1^ To implement the AUDIT in a national context, questions on how best to place it in the national treatment system – including primary and specialized care for alcohol use – need to be answered. Determining the best cut-off scores for different risk levels and the subsequent management of alcohol use disorders is crucial, and validation studies are a standard and required step in such an implementation process.^6^^,^^7^ Although international studies report a wide range of AUDIT cut-off scores for different settings, most of them are not based on validation efforts, which reduces the instrument’s efficiency.^8^

**Fig. 1:**
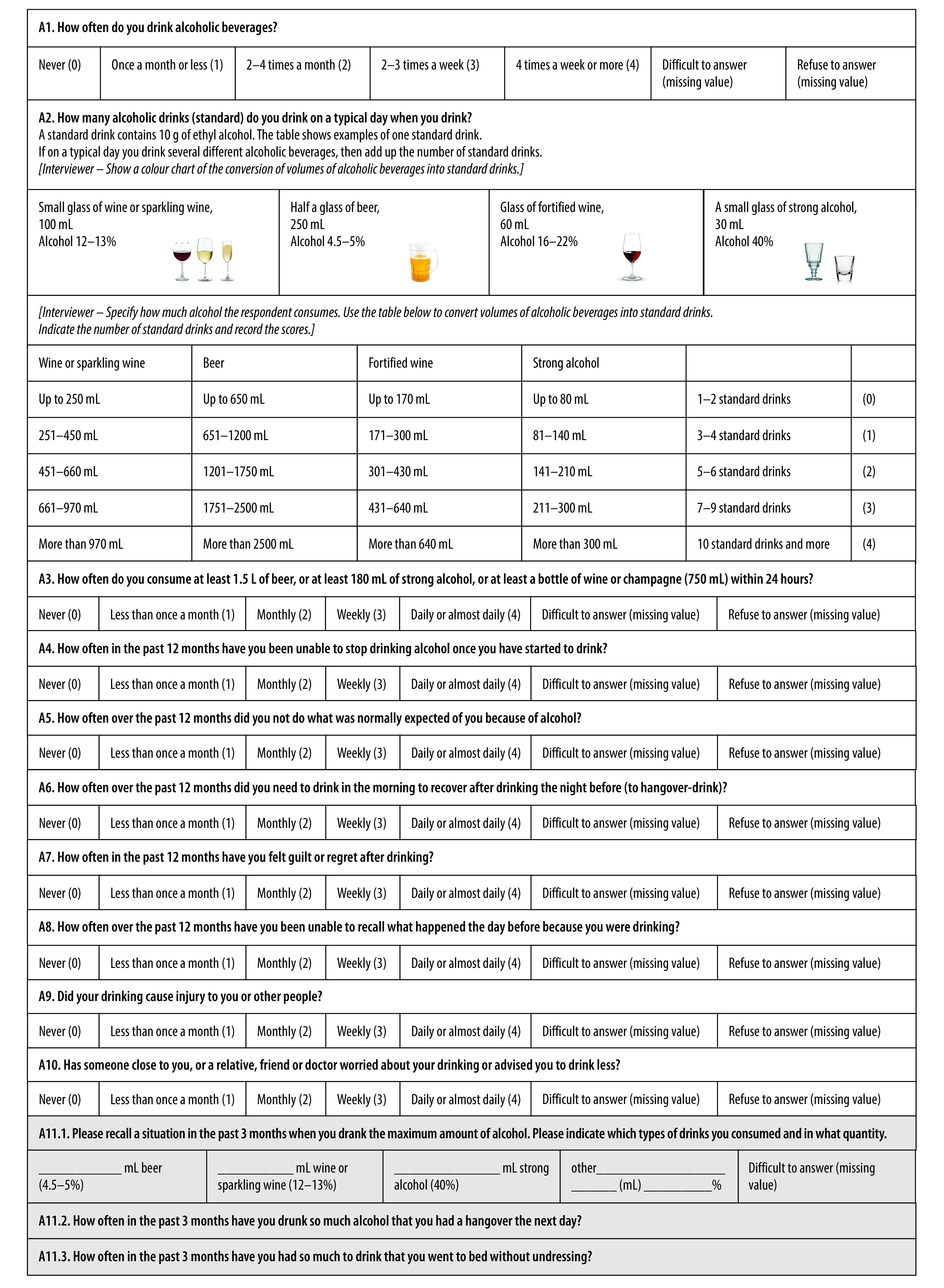
Modified 13-item Russian Alcohol Use Disorders Identification Test for the validation study

In the Russian Federation in 2016, the WHO Regional Office for Europe and the Russian health ministry sought to include the AUDIT as part of an initiative to systematically implement screening and brief interventions.^9^ However, experts involved in the initiative expressed concerns that the AUDIT might not adequately assess the drinking patterns specific to the Russian Federation and its neighbouring countries, mainly because none of the Russian versions of AUDIT had ever been validated.^10^ In addition, issues were found with two terms: the so-called standard drink and single occasion of drinking (intended to assess heavy episodic drinking).^10^^–^^12^ As a result, a Russian AUDIT needed to be revised and validated. Accordingly, we established a protocol for the process and developed a modified AUDIT for use in the Russian Federation, hereafter called the RUS-AUDIT (Fig. 1), details of which are given elsewhere.^13^ Several changes were made to the AUDIT. First, the RUS-AUDIT includes a conversion table of beverage volumes to help interviewers quantify standard drinks as asked about in item 2. Second, the term single occasion in the third item was changed to 24 hours to account for prolonged heavy drinking episodes. Third, three test items about heavy drinking occasions typically reported in the Russian Federation were added: item 11.1 asked for the most alcohol drunk on one occasion in the past 3 months; 11.2 asked about the frequency of hangovers in the past 3 months; and 11.3 asked about the frequency of sleeping without first undressing in the past 3 months. The original score sheet with interviewer codes and instructions are available in the data repository.^14^

RUS-AUDIT: Russian Alcohol Use Disorders Identification Test.

Notes: The last three test items (shaded grey) were initially included in the validation study but are no longer recommended for use in the final RUS-AUDIT version. The test was back-translated into English.

The objectives of our study were to (i) validate the RUS-AUDIT; (ii) empirically examine additional alcohol use patterns (item 3 of the AUDIT) to allow for the best identification of hazardous use patterns in the Russian context; (iii) determine the best cut-off scores for providing brief advice and interventions in primary health-care settings; and (iv) determine the best cut-off scores for potential alcohol use disorders.

## Methods

### Design

We chose concurrent validation as part of a cross-sectional study design to determine how well the Russian AUDIT values correlate with values of other diagnostic tests that have been validated before (see below), and which combinations could serve as measures relevant for future interventions (brief interventions or referral to the specialist treatment system).

### Sample

A total of 21 primary health-care facilities from nine regions, covering seven out of eight Federal Districts of the Russian Federation, participated in the data collection for validation of our RUS-AUDIT (data repository).^14^ We recruited a probability sample of  2173 participants from rural and urban facilities with at least 200 participants from each region. We collected data between August 2019 and February 2020. We established the following quotas for the subsample from each region to secure representation of all important sociodemographic groups:^3^ 50% males,^3^ 50% 40 years and older and not more than 50% recruited from a so-called dispanserization setting. Dispanserization is a term used in the Russian Federation to denote preventive activities undertaken at the population level and organized within primary health-care facilities. These activities include measures such as specialized medical examinations for the early detection of diseases and risk factors, including alcohol use.^15^

The sampling frame was all patients who visited a participating primary health-care facility on the day of the interviews. After providing the patients with medical services, the treating doctors or nurses referred the patients to trained interviewers for the assessment in a separate room.

### Interviewers 

We trained five interview trainers, using modules we had developed and a special training manual for interviewers. These interview trainers carried out nine training sessions for interviewers between August and October 2019 in the participating regions. The training sessions lasted 6–7 hours and covered the basics of screening and brief interventions for alcohol, the structure of the AUDIT, an overview of the RUS-AUDIT validation project, the basics of interviewing techniques, and a thorough overview of the instruments used (data repository).^14^ The trainers used role play to simulate the interview process. At the end of the training sessions, the trainers assessed each trainee individually using a role play of specific interview situations. Only trainees who could demonstrate an ability to administer the interviews correctly were selected as interviewers by the trainers.

### Interview procedure

**Fig. 2:**
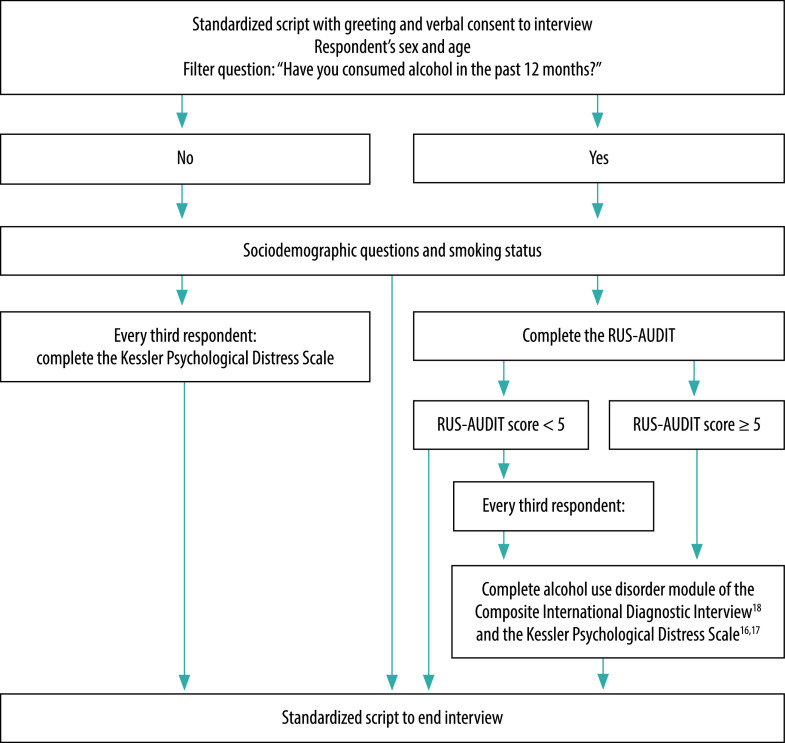
Flowchart of the interview process for the validation of the Russian Alcohol Use Disorders Identification Test

Participants provided verbal consent before being interviewed using a standardized form. The form included questions on demographic characteristics (age, sex, type of housing and disposable income) and drinking and smoking status. Participants who had consumed alcohol within the past 12 months were further interviewed using the modified 13-item version of the RUS-AUDIT. All participants with a RUS-AUDIT score of five and higher and thus indicating a certain risk level were then asked to complete the Kessler Psychological Distress Scale^16^^,^^17^ and the alcohol use disorder module of the Composite International Diagnostic Interview.^18^ Both instruments were translated into Russian according to WHO guidelines.^19^ We also asked a subsample of people with AUDIT scores lower than five (every third person) to complete these instruments (Fig. 2).^14^ In addition, we gave the Kessler instrument to every third person who had not consumed alcohol in the past 12 months to have an additional control variable; we did not give it to all such participants to save on assessment time as the Kessler scale applies to mental distress and not just alcohol use disorders. The institutions participating in the study undertook quality control procedures for the interviews in their institutions by checking each form directly after completion and checking all forms before submitting.

RUS-AUDIT: Russian Alcohol Use Disorders Identification Test.

### Statistical procedures

The first step was a descriptive statistical analysis of the study sample, defining the final analytic sample size for the RUS-AUDIT validation study and visually examining the score distribution. Next, we examined one-dimensionality and internal consistency of the RUS-AUDIT scale by calculating the Cronbach *α*  and other internal consistency measures. We carried out additional analyses to determine whether any of the three additional items on heavy drinking (11.1, 11.2 and 11.3; Fig. 1), would improve the psychometric properties. We performed receiver operating characteristic analyses to test whether the RUS-AUDIT had acceptable statistical properties to predict four main outcomes: hazardous drinking, problem drinking, harmful use and alcohol dependence. 

Hazardous drinking was defined as an alcohol intake of > 20 g/day for women and > 40 g/day for men, based on the drinking categories identified by WHO and the European Medicines Agency in the absence of low drinking guidelines in the Russian Federation.^20^ We introduced problem drinking as an operational definition to denote the next relevant risk level as per the RUS-AUDIT scale and based on scoring on any of the following test items of the Composite International Diagnostic Interview: health problems related to drinking; objections by family or friends to drinking; collapse of relationship with loved ones due to drinking; financial difficulties due to drinking; attacked or injured someone while intoxicated; problems with police (drink–driving, accident); reduced time for important activities (work or leisure); and have had a disease (e.g. liver disease, stomach problems) or psychological problems (depression, anxiety) due to drinking.^18^ These questions correspond with broader problems associated with alcohol use and some symptoms of alcohol use disorder, partly meeting the ICD-10 criteria.^4^ We defined harmful alcohol use and alcohol dependence according to the relevant items of the Composite International Diagnostic Interview, following the ICD-10 classification, which is the manual currently used to diagnose alcohol use disorders in the Russian Federation.

As for the cut-offs, we selected the two outcomes linked to secondary prevention (hazardous and problem drinking) based on the Youden index, which is the difference between the proportion of true-positive and false-positive results.^21^ We selected the criteria for requiring a treatment intervention (alcohol dependence alone and alcohol use disorders, which includes both harmful use and alcohol dependence) based on accuracy and specificity and in case of equal values on accuracy to avoid unnecessary costs to the health-care system and potential registration in a treatment programme.

To identify the best short version, we tested all possible combinations of three (a total of 286 combinations are possible) of the 13 RUS-AUDIT items for prediction of the full RUS-AUDIT score and for prediction of the four main outcomes specified earlier. For this exercise, we ran two alternative analyses: in the first, only the original items 1–10 were included, while in the second, we added the alternative items 11.1, 11.2 and 11.3. 

### Ethical considerations

Since this study's main goal was one of quality improvement in the participating primary health-care facilities without collecting any identifying patient information, it was considered to be part of routine care by participating institutions, except for the specialized addiction care centre, where it underwent ethical review (this is similar to other implementation studies for screening and brief advice/interventions in other countries^22^). Even though the study was one of quality control, we asked for verbal consent from patients willing to participate.

All parts of the study, including pilots and pre-studies, were fully compliant with ethical principles, including the provisions of the World Medical Association Declaration of Helsinki, as amended by the 59th General Assembly, Seoul, the Republic of Korea.

## Results

### Sample

Of 2173 people approached to join the study, 143 (6.6%) declined to participate. Of the remaining 2030 people, two did not fall within the required age range and six did not identify their sex. Therefore, 2022 people (1036 men and 986 women) made up the sample for the RUS-AUDIT validation study and answered the questions on sex, age and alcohol consumption. Of the 2022, 1513 (74.8%) reported alcohol use in the past year, of whom 1497 (809 men and 688 women) provided valid RUS-AUDIT responses, i.e. 98.9% of the completed forms had no missing values. More information on the sample is available in the data repository.^14^

### Psychometric properties

The RUS-AUDIT proved to be easy to administer and had few missing values as outlined earlier. Internal consistency of the RUS-AUDIT was good (Cronbach *α* : 0.842). All items contributed to the scale and the removal of any item resulted in a lower Cronbach *α* value. Principal component analyses showed that the first item of the test explained 49.1% of the variance and none of the factor loadings were lower than 0.594. The RUS-AUDIT had good psychometric properties for both sexes.

We found no increase in internal consistency when any of the three items (11.1, 11.2, 11.3) replaced the current item 3 of the RUS-AUDIT on drinking patterns (alternative Cronbach *α*  scores were 0.833, 0.840 and 0.830, respectively).

**Fig. 3:**
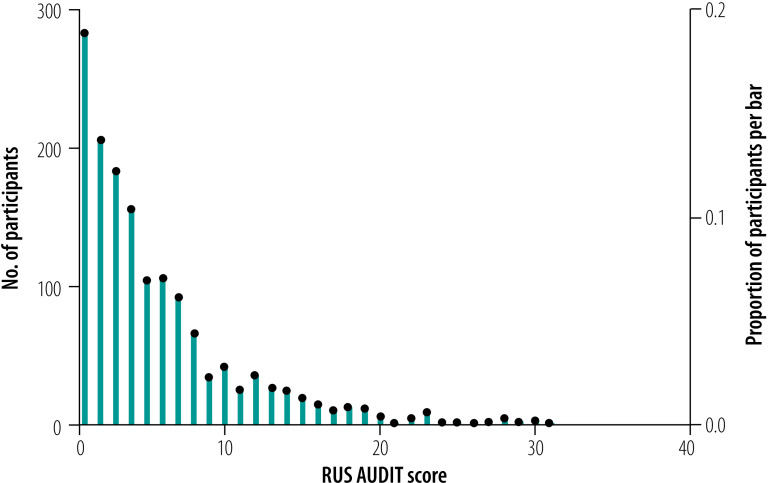
Population distribution of scores on the Russian Alcohol Use Disorders Identification Test among people who drink alcohol

The distribution of the RUS-AUDIT among drinkers is shown in Fig. 3. As with level of alcohol use,^23^^,^^24^ we found a typical gamma distribution with a peak on the left-hand side and a fairly long tail to the right-hand side. The overall mean score in the RUS-AUDIT was 5.76 (standard deviation, SD: 5.50), the mode was 1 and the median 4. Women had significantly lower scores (mean: 3.40; SD: 3.54) than men (mean: 7.78; SD: 6.05; *P* < 0.001). 

RUS-AUDIT: Russian Alcohol Use Disorders Identification Test.

Note: *n* = 1497.

### Prediction of main outcomes 

Table 1 shows the best thresholds and prediction characteristics of the RUS-AUDIT to predict the main outcomes (hazardous drinking, problem drinking, alcohol use disorders and alcohol dependence) necessary to set up treatment.

**Table 1:** Prediction characteristics for the main outcomes in the Russian Alcohol Use Disorders Identification Test

Outcome (criterion for selection)	Threshold score on RUS-AUDIT			Area under the curve (95% CI)			Correctly classified, no. (%)^a^			Sensitivity, %			Specificity, %	
	Women	Men		Women	Men		Women	Men		Women	Men		Women	Men
Hazardous drinking^b^	5	9		0.964 (0.926–1.000)	0.919 (0.892–0.946)		543 (78.9)	596 (73.7)		100.0	98.2		78.6	71.8
Problem drinking^b^	6	10		0.831 (0.772–0.890)	0.857 (0.828–0.887)		301 (78.8)	476 (78.7)		74.3	67.3		79.9	89.4
Alcohol use disorders^c^ (as defined in ICD-10)^4^	10	14		0.872 (0.820–0.925)	0.838 (0.805–0.872)		348 (91.1)	488 (80.7)		48.3	51.0		99.1	94.4
Alcohol dependence^c^ (as defined in ICD-10)^4^	11	17		0.936 (0.904–0.967)	0.879 (0.844–0.913)		360 (94.2)	528 (87.3)		57.6	47.6		97.7	97.1

CI: confidence interval; ICD-10: *International statistical classification of diseases and related health problems, 10th revision*; RUS-AUDIT: Russian Alcohol Use Disorders Identification Test.

^a^ For hazardous drinking, *n* = 1497 (688 women, 809 men); for the other three criteria, *n* = 987 (382 women, 605 men).

^b^ Selected based on Youden.^21^

^c^ Selected based on accuracy and specificity.

Overall, the RUS-AUDIT predicted all outcomes well, and was slightly more accurate for women than for men based on receiver operating characteristic analyses. The area under the curve was greater than 0.83 for all outcomes, and between 74% and 94% of the patient outcome scores were correctly predicted using the proposed algorithms of classification. Sex-specific thresholds were necessary for all outcomes: using non-specific thresholds resulted in very few women being identified with any outcome.

### Best short versions

For predicting the RUS-AUDIT, the combination of items 3, 9 and 10 was the strongest. We will call this combination the RUS-AUDIT-S. The Pearson correlation coefficient (*r*) with the full RUS-AUDIT was 0.923 (95% confidence interval, CI: 0.916–0.931) with 85.3% of the variance explained. This combination was significantly better than the commonly used AUDIT-C (items 1, 2 and 3), which had a correlation coefficient of 0.862 with 74.4% of the variance explained (*P* < 0.001). The RUS-AUDIT-S also performed significantly better than the AUDIT-C when scores for only women or men were included separately: *r*: 0.894 (95% CI: 0.878–0.908) compared with *r*: 0.853 for the AUDIT-C (*P* < 0.001) for women; and *r*: 0.915 (95% CI: 0.903–0.925) compared with *r*: 0.835 (*P* < 0.01) for men. 

The RUS-AUDIT-S was the best average predictor of all of the outcomes tested: hazardous drinking, problem drinking, alcohol use disorders and alcohol dependence as defined by the European Medicines Agency^20^ and the ICD-10;^4^ alcohol dependence or alcohol abuse as defined by the *Diagnostic and statistical manual of mental disorders*, fourth edition (DSM-IV);^25^ or alcohol use disorders as defined by the DSM-V.^26^ For all outcomes, the area under the curve was greater than 0.80 (for point estimates, please see data repository).^14^ This finding means that some outcomes were even better predicted by the three-item RUS-AUDIT-S than by the full 10-item RUS-AUDIT.

Another combination (items 1, 10 and 11) was an even better average predictor of the outcomes tested. However, as this combination included item 11 which was not selected for inclusion in the final version of the RUS-AUDIT, the combination of items 3, 9 and 10 was kept as the standard short version of the RUS-AUDIT.

As there had previously been an AUDIT-4 in use in the Russian Federation (a short version consisting of the first three and the last AUDIT test items),^27^ we also tested all 715 combinations of four items for the best correlation with the outcomes (data repository).^14^ The AUDIT-4 combination scored worse on all of the outcomes than the combination selected as the best average combination of the RUS-AUDIT-S (data repository).^14^ Thus, if a four-item combination is to be used, the most suitable combination of RUS-AUDIT items is items 1, 3, 9 and 10. The three-item combination of the RUS-AUDIT-S, however, produced similar statistical properties for most outcomes and, because it is shorter, is preferable for use in primary health care.

## Discussion

The RUS-AUDIT was internally consistent, capable of predicting hazardous drinking, problem drinking and alcohol use disorders, and feasible for use in primary care settings in the Russian Federation. Nonetheless, some potential limitations exist.

First, while we had good representation of different regions of the Russian Federation and established probability samples within each primary care facility, our sample may not be statistically representative of Russian primary health-care patients. However, our non-response rate was low, indicating that non-response rates did not pose a problem. Second, the study relied on self-reporting by the participants and, while all of the study instruments had been validated against non-self-reported gold standards in the past (e.g. AUDIT^28^ and the Composite International Diagnostic Interview^29^ were validated against non-self-reports in the past), we cannot exclude the possibility that bias is introduced in such assessments. Although comparing the outcomes of one instrument (AUDIT) with a validated gold standard (Composite International Diagnostic Interview and Kessler Psychological Distress Scale) is a standard practice in concurrent validation, there are limitations of self-reports such as social desirability or recall bias, especially given the similarity and cross-sectional nature of the data collection methods. We could only have removed this limitation with the introduction of reliable and valid external criteria, such as a professional examination and diagnosis of each participant by an addiction specialist. However, this course of action would have required additional resources.

Our results clearly show distinct patterns of drinking in the Russian context. The first three consumption items were less important to the total score than in other countries.^3^^,^^6^ In fact, the average level of drinking a day was relatively low, and a lot of the alcohol was consumed on heavy drinking occasions, which had higher volumes of alcohol consumed per occasion than in countries with the same overall level of consumption, e.g. Germany.^30^ This finding has two implications that need to be considered when implementing the RUS-AUDIT and when comparing it to other versions of the AUDIT. First, this finding means that many concepts of high-income countries in western Europe and North America that centre around average heavy drinking over time^31^ do not necessarily apply in the Russian Federation. Second, the low contribution of the first three consumption items to the final test result leads to relatively low thresholds for the relevant outcomes when compared with other versions of the AUDIT.^3^^,^^6^^,^^7^^,^^32^

However, our attempts to replace the third item of the AUDIT with other items (11.1, 11.2 and 11.3) found relevant in other studies in the Russian Federation^33^ showed no improvement in internal consistency. Nonetheless, these items did contribute to predicting negative outcomes and appeared in several combinations to predict problem drinking and alcohol use disorders. Thus, as long as Russian drinking patterns continue to be distinct from drinking patterns of other middle- and high-income countries, we recommend retaining these items in any scientific studies on drinking conducted in the Russian Federation.

The background to our study was the implementation of screening and brief interventions in primary health care in the Russian Federation. The RUS-AUDIT and the RUS-AUDIT-S, which reduces costs while still effectively identifying people with hazardous and harmful drinking patterns, could be used for such screening. This more individualized approach of screening and brief interventions aimed at reducing alcohol-attributable harm could complement the current population approaches in the Russian Federation, which have proven successful to date,^34^^,^^35^ thus achieving application of all elements of the WHO SAFER initiative.^1^ The use of RUS-AUDIT and RUS-AUDIT-S in a digital format, possibly as a mobile application, could be a promising new approach to screening as it would reduce the time required by a primary health-care worker to administer the instrument. A digital format may even increase accuracy due to more flexible and intuitive ways of assessing alcohol use through animated elements and the automatic calculation of standard drinks by the application.

The challenge of implementing the RUS-AUDIT within the health-care system will be to properly define what is possible in which parts of the primary health-care system, and under what circumstances people should be transferred to the specialized alcohol and drug treatment system. According to Russian legislation, specialized treatment is provided free of charge, but individuals are officially registered as so-called narcological patients once diagnosed and a prolonged monitoring regimen is imposed. These patients are automatically excluded from certain professions, can lose their driving licence and may experience social stigma and discrimination because of their status. Therefore, official registration is often a punitive experience and can be an important barrier to help-seeking behaviour.^34^^,^^36^ Thus, mandatory registration should be re-examined in the interests of establishing a safe and high-quality screening and care system in the country. The main goal will be to create a system of health care that recognizes the importance of early intervention and prevents hazardous consumption and severe alcohol use disorders from becoming more chronic,^37^ and thereby avoids the associated attributable harm.^38^ A suggestion on how the system could be designed using the RUS-AUDIT and the RUS-AUDIT-S is discussed in the data repository.^14^

The RUS-AUDIT is the first rigorously translated and adapted Russian version of the AUDIT and it potentially provides an additional high-impact strategy for Russian alcohol control policies, complementing the existing successful general population strategies.^34^^,^^35^ Furthermore, its potential for use for Russian-speaking populations with similar drinking patterns outside of the Russian Federation should be explored. 

The experience of developing and validating the RUS-AUDIT shows that translating standardized instruments such as the AUDIT into other languages may not provide a reliable tool without the introduction of modifications and prior rigorous and culture-specific research and evaluation. We hope that the documented experiences and materials developed^11^^–^^13^ can be useful for other countries as models for validation procedures.
